# Relationship between Night Shifts and Risk of Breast Cancer among Nurses: A Systematic Review

**DOI:** 10.3390/medicina56120680

**Published:** 2020-12-10

**Authors:** Javier Fagundo-Rivera, Juan Gómez-Salgado, Juan Jesús García-Iglesias, Carlos Gómez-Salgado, Selena Camacho-Martín, Carlos Ruiz-Frutos

**Affiliations:** 1 Health Sciences Doctorate Programme, University of Huelva, 21007 Huelva, Spain; javier.fagundo308@alu.uhu.es (J.F.-R.); cargomsal@gmail.com (C.G.-S.); 2 Department of Sociology, Social Work and Public Health, Faculty of Labour Sciences, University of Huelva, 21007 Huelva, Spain; juanjesus.garcia@dstso.uhu.es (J.J.G.-I.); frutos@uhu.es (C.R.-F.); 3 Safety and Health Postgraduate Programme, Universidad Espíritu Santo, 092301 Guayaquil, Ecuador; 4 Fundación Andaluza Beturia para la Investigación en Salud, University Hospital Juan Ramón Jiménez, 21005 Huelva, Spain; selenacm22@gmail.com

**Keywords:** shift work schedule, night shift work, breast cancer, nurses, environmental, circadian rhythm, occupational exposure

## Abstract

*Background and objectives:* The incidence of breast cancer worldwide has increased in recent decades, accounting for 1 in 3 neoplasms in women. Besides, nurses are mainly represented by the female collective, most of them, undertaking working conditions with intensive rotative and night shifts due to the 24-h pace of work of this profession. The objective of this study was to assess the possible relationship between shift work, especially night-time work, and the development of breast cancer among nurses. *Materials and Methods:* A systematic review of the literature was carried out through the consultation of the following databases: Cochrane Plus Library, PubMed, CINAHL (Cumulative Index to Nursing and Allied Health Literature), Web of Science, ScienceDirect, Scopus, and Dialnet. Records were selected between 2010 and 2020, in Spanish and English, which covered the association between breast cancer diagnosed among nursing professionals and rotating night shifts. *Results:* A total of 12 studies were identified after critical reading. Most of the studies found an association between breast cancer and consecutive rotating night shifts prolonged over time. Among the associated factors, the alteration of the circadian rhythm influenced the expression of peripheral clock genes, which was the same as reproductive hormones. The risk of breast cancer in nurses increased during early adulthood and after 5 or more years with 6 or more consecutive nights. *Conclusions:* The different studies of this review show significant associations between breast cancer and prolonged rotating night shifts. Similarly, there is a relationship between the alterations in certain circadian rhythm markers (such as melatonin), epigenetic markers (such as telomeres), and breast cancer that would require more studies in order to corroborate these findings.

## 1. Introduction

Breast cancer currently represents the neoplasm that causes the highest incidence and death among women. This type of neoplasms usually presents itself as a painless mass in one or both breasts, where the help of complementary exploratory tests is necessary for diagnosis, such as breast imaging tests (mammography) as proof of choice, or breast biopsies if the image suggests malignancy, which allows a histological diagnosis and an immune-histochemistry study. Its prognosis is related to the biological subtype and tumor stage of the patient. Treatments will vary depending on the characteristics and location of the tumor. Systemic chemotherapy treatments may be combined, if necessary, with other local treatments such as radiation therapy and surgery, depending on the situation. If metastasis is diagnosed, treatment seeks to improve or maintain the quality of life and increase the expectancy of survival of the person, having a palliative character. Although the knowledge of its etiology in recent years has made substantial progress, strongly relating it to multiple factors such as aging, unhealthy lifestyles, or changes in reproductive patterns, there are few revelations regarding occupational risk factors [1,2,3].

Human beings, like all species on our planet, have developed their biochemical and physiological processes such as cell cycle, apoptosis, genetic expression, or hormonal secretion around the 24-h rhythm marked by Earth’s rotation around the Sun, which is organized in daily periods where there is exposure to light, from dawn to dusk, and to darkness. Shift work alters this organization by breaking the circadian rhythm governed by the endogenous clock found in the suprachiasmatic nucleus of the hypothalamus, belonging to the Central Nervous System, as well as altering dietary patterns, among other processes [4,5]. In this sense, one of the most evaluated risk factors in recent years has been night work for its impact on the circadian cycle, ranked in 2007 by the International Agency for Research on Cancer (IARC) as a likely carcinogenic factor for humans (Group 2A), based on limited evidence from epidemiological studies and sufficient evidence from animal models. To date, this agency has evaluated more than 900 agents and/or types of exposures to establish whether they are carcinogenic for humans. It has also been hypothesized that night work can pose a risk factor due to sleep deprivation, decreased melatonin hormone due to exposure to light at night, or modulation of sex hormones among others [3,4,6,7,8,9,10,11,12,13].

People doing night shift work are currently increasing around the world, especially in industrialized countries. This is because more and more regions of the world are adopting the 24-h-a-day model of society, both at home and at work, exposing the population, and especially the female collective, to more hours of artificial light [2,14].

This is clearly seen in a profession such as nursing, where nurses base their work organization on shifts in order to ensure the highest quality of care through continuity of care for patients 24 h a day. This represents higher levels of stress and, therefore, an increase in the number of psychological and physiological problems in this role, thereby increasing the number of casualties (with about 60% of the working days lost) and causing an annual cost of around 20 billion euros. To this we must add the increased risk of making mistakes during the shift and, therefore, a loss in the quality of care [15]. The nursing profession has been highly studied in this regard, mostly with epidemiologic studies, exposing a relationship between prolonged night work and an incidence of between 50% and 100% of breast cancer cases [16].

Therefore, it is necessary to understand the mechanics of these processes and the impact these generate on the nursing professional in order to provide conclusions that allow to take the necessary preventive measures to minimize the risk of breast cancer. In this manner, the objective of this study is to evaluate the possible relationship between shift work, especially night-time work, and the development of breast cancer among nurses.

## 2. Materials and Methods

### 2.1. Design

A systematic review of the records of different databases was carried out, following a pre-set protocol initially established to minimize the risk of bias in both choice and publication, thus ensuring optimal organization and content, all through the application of the standards set out in the PRISMA declaration [17]. Using the Evidence-Based Health Practice (PSBE, for its acronym in Spanish) methodology [18], and the PICO format (Patient/Problem/Population, Intervention/Indication, Comparison, Outcome) (Table 1), the clinical question was developed. After the first scientific evidence screening, the FLC 3.0 web platform [19] is utilized for the critical review of the chosen references. This revision protocol has been recorded in PROSPERO with registration number CRD42020216309.

Based on the information detailed in Table 1, the following question was asked: “Is there a link between breast cancer and night work among nurses?”

### 2.2. Databases and Documentary Sources Consulted

The bibliographic search took place between 7 July 2020 and 27 July 2020. This review was carried out in the following databases:

- Cochrane Plus Library
- PubMed
- CINAHL (Cumulative Index to Nursing and Allied Health Literature)
- Web of Science
- ScienceDirect
- Scopus
- Dialnet

### 2.3. Keywords

In the search, keywords obtained from the Descriptors in Health Sciences (DeCS) and Medical Subject Headings (MeSH), developed by the National Library of Medicine (NLM), were used depending on the language used in the database. Details of the different search strategies used are displayed in Table S1. The keywords included in the search terms are detailed by language in the following table (Table 2):

### 2.4. Inclusion and Exclusion Criteria

The inclusion and exclusion criteria were as follows:

Inclusion criteria:

- Studies carried out over the last 10 years (2010–2020).
- In Spanish and English.
- Peer-reviewed articles.
- Typology: original articles and clinical trials, systematic meta-analysis and reviews, short/brief communication, and case reporting.
- They should analyze at least one of the following characteristics on a nursing sample (population): risk factors related to shift work among nursing professionals and breast cancer, associated hormonal changes and/or alterations after blood sample, light levels to which nurses are exposed at night work, and circadian gene expression.

Exclusion criteria:

- Records of low scientific evidence.
- Articles that have no relation to the purpose of the review.
- Typology: opinion articles, editorials, and letters to the director/editor.

The inclusion criteria set out above were used as a template in the first screening to which all selected studies were submitted. Those who exceeded all the criteria were given a positive sign (+) and those who did not, a negative one (−), thus being excluded. The inclusion criteria list can be seen in Table 3 [2,3,4,5,6,7,8,9,10,11,12,13,14,15,16,20,21,22,23,24,25,26,27,28,29].

### 2.5. Critical Appraisal and Level of Evidence

For the critical appraisal of the bibliography, the FLC 3.0 web platform for Critical Reading Sheets (Osteba: Pais Vasco, Spain) [19] was used, a tool for assessing study quality developed by the Health Technology Assessment Service of the Basque Government. This tool uses the recognized evaluation criteria according to the study design that evaluates each sheet, and thus assesses the methodological quality of each study and the degree of bias in the different methodological designs, facilitating criteria homogeneity among reviewers. Both the quality assessment and data extraction were independently carried out between pairs, and a third party acted as an evaluator comparing the information collected between both evaluators and agreeing on the contents of the final template.

The selected records were assessed following the following points below:

- Author.
- Date.
- Study design, objective, location, and period of completion.
- Study population, intervention/comparison, analyzed results, and follow-up time.
- Number of participants, intervention in experimental and control groups, masking method, and post-randomization losses.
- Results, beneficial and adverse clinical effects.
- Conclusions.
- Study quality.

Once this section had been complied with, the level of quality of scientific evidence of each of the studies was subjectively determined, and a classification with a low, medium or high level was be obtained. After this process, studies that obtained a low-quality level of evidence were excluded (Table 4).

### 2.6. Reverse Search

As a secondary strategy, a search was carried out using references and author names cited in the different selected records (reverse or snowball search), with the intention of verifying the existence of works not found in the primary search.

## 3. Results

### 3.1. Records’ Selection

**Figure 1:**
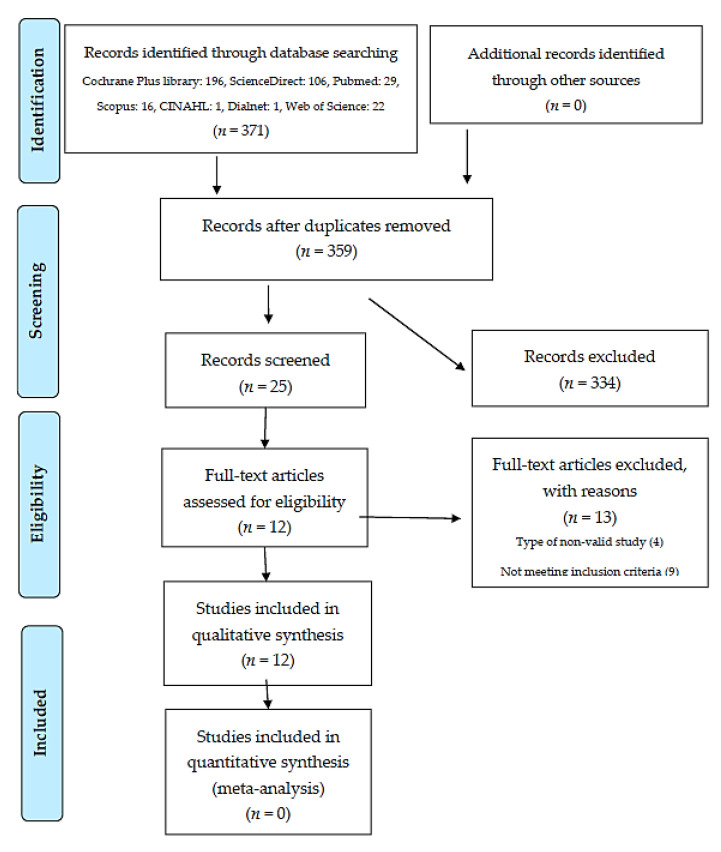
Bibliographic search flowchart adapted according to the PRISMA model (Preferred Reporting Items for Systematic reviews and Meta-Analyses) [17].

The total number of records found in the different databases was 371, of which 37 were selected after a first screening where studies that did not provide relevant data to the work, after reading the title and summary, were discarded. Of these, 25 studies were kept after the removal of duplicates. Once the evaluation of these studies was carried out, those that met the pre-established inclusion criteria were selected, with a total of 12 records meeting these requirements (see Table 3). Following this process, and as the last phase of selection, these 12 studies met the quality criteria [19] established in the critical reading phase [2,4,5,6,7,8,9,13,14,15,21,29] (Table 4). The flowchart for this study is presented in Figure 1.

### 3.2. Summary of the Studies

Table 5 [2,4,5,6,7,8,9,13,14,15,21,29] summarizes the evaluated aspects of the 12 selected studies: main study characteristics, aim of the study, intervention and instrument, main findings, conclusions, and quality assessment for each of the records.

## 4. Discussion

The association between breast cancer and rotating night shifts among nurses is a constant in most of the examined studies in this review. The majority of studies reported a relation between increased breast cancer risk and cumulative years working in night shifts, normally with 3 or more nights per month for 15 years or more [2,4,5,6,7,8,9,13,14,15,29]. Risk increases in permanent night shifts and long-term day–night rotating shifts [2]. A long duration of shift work throughout years is also related with estrogen and progesterone positive tumors, mostly found among young women with intensive shifts (12 h-shifts) [8,9,14,15,21]. Most of the studies add information that relates rotating shift work and lifestyle factors [2,4,5,6,7,8,9,13,14,15,21,29], being quality of sleep, obesity, diabetes, early menopause, number of childbirths, and hormonal treatment the most common factors related with night work. However, the relationship between rotating night work and the expression of circadian genes [5,6,8,13,21], as well as the relationship between certain markers of circadian rhythm and genetic alteration or expression [7,13,21,29], continues to be discussed.

Wegrzyn et al. [14] examined, on two prospective cohorts, Nurses’ Health Study (NHS: 1988–2012, sample: 78,516), and Nurses’ Health Study II (NHS2: 1989–2013, sample: 114,559), with 9541 cases of invasive breast malignancies and 24 years of follow-up. In the NHS, women with more than 30 years of shift work did not have an increased risk of breast cancer (Risk Ratio (RR)—0.95 (95%); CI 95% = 0.77–1.17; *p* = 0.63), as compared to those who never worked in shifts, although the follow-up was mainly carried out after the women finished doing shift work. However, at NHS2, there was a significant increase in the risk of breast cancer among nurses with more than 20 years of work, reflecting exposure among young adult nurses (RR 2.15; CI 95% = 1.23–3.73; *p* = 0.23) and significantly increasing among those nurses who had been performing cumulative rotating night shifts for more than 20 years since their early career, updating the exposure information (RR 1.40; CI 95%, 1.00–1.97; *p* = 0.74). Thus, associations between rotating night shifts work and the risk of breast cancer concluded that rotating night work, sustained over time, increased the risk of breast cancer among those women who performed these shifts at an early age.

The study conducted by Lie et al. [6] in 2011 on a cohort of 49,402 Norwegian nurses found a total of 699 (74%) of cases diagnosed between 1990–2007, and alive at the moment of the study, and 895 (65%) cancer-free cases at the time of the sampling. This study assessed the association between breast cancer and night work, and suggested that these results could establish a link between the risk of breast cancer and the number of consecutive shifts, finding a significantly higher increase in breast cancer risk, between 10–30%, among those professionals with a higher level of exposure and establishing that those nurses who had worked 5 years with 6 consecutive night shifts (OR 1.8; 95% CI: 1.1–2.8) had an increased risk of developing the disease, as compared to those who had never worked on the night shift. In later studies, a number of polymorphisms was found in circadian genes and melatonin biosynthesis that could be associated with the risk of breast cancer among nurses working on shifts [30,31]. Hansen et al. [4], in another case and control study, conducted in 2011 based on interviews to a national cohort of 91,140 women, members of the Danish Association of Nurses (95% of nurses in Denmark), also exposed an increasing trend in the increase in cases of breast cancer related to cumulative shifts work with greater or lesser risk to alter the circadian rhythm.

Although the number of studies included in the systematic review conducted by Dickerman et al. [2] in 2012 was somewhat limited, its authors also explained that exposure to light during periods of night work may contribute to an increased risk of breast cancer among nurses, analyzing potential action mechanisms associated with night work such as melatonin suppression, sleep disruption, lifestyle factors (poor diet and exercise, higher body mass index) or lower levels of vitamin D. Similarly, the characteristics of night work were significantly associated with alterations in bodily hormones such as oestradiol [9,21] or insulin [15]. Some studies have strengthened the relationship between urinary levels of 6-sulfatoxymelatonin (aMT6s) and shift work, a stable metabolite of urinary melatonin that appears to be related to breast cancer [15,28]. Moreover, the release of luteinizing hormone (LH) and follicle stimulating hormone (FSH) by the pineal gland, which stimulates the production and release of oestrogen by the ovaries, was associated with melatonin levels, so it can be concluded that the decrease in circulating melatonin concentration could give rise to a greater amount of oestrogen secreted by the ovaries [15]. For its part, the case and control study carried out by Lie et al. [8] in 2013 assessed the effect of night work on diagnosed breast cancer nurses between 1996–2007 on 757 controls that were selected from a nurse cohort in Norway. The study offered statistically significant data on the impact that long-term night work (≥5 years) with 6 or more consecutive shifts have on the increased risk of breast cancer, noting significant associations and an increased risk for tumors with positive progesterone receptors (OR = 2.4, CI 95%: 1.3–4.3; *p* = 0.01). These findings suggested an important effect of progesterone on the negative impact that shift work has among nursing professionals. In line with this finding, the Carugno et al. [29] study observed a reduction in methylation of the ESR1 gene (mediated by linking progesterone to the promoter region) associated with night shift work, both by analyzing current night shift workers versus non-night shift workers, and by comparing those who never worked on the night shift, suggesting that low levels of ESR1 were associated with increased proliferation of breast tissue.

Erdem et al. [7], in his 2017 study, referred that telomere length (TL) shortening was associated with an increased risk of breast cancer among workers who had performed long periods of consecutive night shifts, evaluating quantitative polymerase chain reaction (qPCR) in the DNA of 563 patients diagnosed with breast cancer and 619 Norwegian control nurses. The data indicated that TL was not significantly different among nurses who had worked night shifts as compared to those who had worked only on isolated days, concluding that the duration of night work, whichever the night shift intensity, did not influence TL. On the contrary, it was noted that working many consecutive night shifts for at least 5 years had a correlation with a reduction in TL regardless of the state of the control cases. Another study based on the genetic study concluded the same when working night shifts for at least 12 years [29]. These data suggest that shortened TL is affected by night work schedules and could be a contributing factor to the risk of breast cancer among women working shifts.

The systematic review carried out by Rosa et al. [15] in 2019 showed data suggesting a link between night shift work and disruption of sleep rhythm among nurses, noting that some of the studies showed that sleep reduction was associated with an increased risk of breast cancer, and also finding an association between breast cancer, shift work, and the number of consecutive night shifts performed over a period of more than 1 month. In this case, the risk of breast cancer was significantly higher (OR = 1.8; CI 95% = 1.2–2.8; *p* = < 0.05) among nursing professionals who had worked for more than 5 years with 6 continued rounds on night shifts. It was stated that the dehydration effects caused by night work were derived from the secretion of cortisol and that, in addition, the functioning of the hypothalamic-pituitary-adrenal axis was affected by lack of sleep hours and deficit daytime rest, contributing to the mismatch of the immune system and impacting on the effectiveness of the anti-tumor surveillance system [15]. It was noted that nurses working night shifts for a period of 1 to 29 years had an 8% risk of developing breast cancer. Meanwhile, nurses who had worked night shifts for more than 30 years showed a higher risk of up to 36%. Among the signs of circadian rhythm disturbance, the review by Rosa et al. [15] also found an association between the increase in irregular menstrual cycles and night shift workers, this being considered a risk factor for breast cancer. Similarly, the review carried out by Vega-Escaño et al. [32] described shift work (regardless of its characteristics and context) with a direct relationship for the onset of insomnia, which could lead workers to be extremely exposed to risks arising from their professional activity, in addition to other factors such as stress, self-medication, tobacco abuse, or the use of psychoactive substances.

Some limitations found in this study were as follows: (a) having used specific search tools (Cochrane, PubMed, CINAHL, Web of Science, Science Direct, Scopus, and Dialnet), so there is a possibility of lost research elements; (b) studies written in English and Spanish from 2010 to the present day were taken into account, excluding the possibility of prior research or research in other languages; (c) some research had a small number of participants and did not meet the objectives of this study after the first critical reading; (d) despite avoiding article selection bias, in order to focus the analysis on breast cancer and shift work schedules among nurses, the inclusion and exclusion criteria limited the review field by excluding low-quality research, other cancers or other professionals; (e) in most of the analyzed research, there are many confounding characteristics among the samples being analyzed, so this should be considered generalizing the findings to the entire nursing population; (f) performing a meta-analysis was not established as an objective of this study. Moreover, heterogeneity has been observed among the selected studies in terms of the analyzed variables, sample size, definition of shiftwork, and analytic methods, so meta-analysis was not employed in a further step.

The results of the study can be valid, indirectly, for all night shift workers apart from healthcare. This is, industrial, transport, communications, leisure, and hospitality sectors. As future measures, analyzing breast cancer risk factors as compared with occupational factors would be necessary to correlate the molecular mechanisms of cells’ circadian control, specifically in the event of circadian disruption during work schedules. For this, a multivariate examination with regression models is projected to understand the entire complexity of shift work, in which the role of moderating/mediating factors remains unexplored. This analysis would be useful to propose preventive measures such as a healthy methods of shift rotation (mostly with reduction of night shifts), developing labor risk stratification algorithms, or assessing the behavioral consequences of different types of rotation.

## 5. Conclusions

The different studies of this review showed a significant relation between breast cancer and prolonged rotating night shifts in the established time. In this way, cumulative years working at night, long shift length (12 h), and performing more than 6 night shifts per month for at least 5 years or more are found as a potential breast cancer risk factors, especially in hormone-dependent cancers and among nurses who started working at night in their early career. Similarly, there is a relationship between alterations in certain markers of circadian rhythm such as melatonin or in markers of epigenetic alteration such as telomeres length and breast cancer, that would require further studies in order to support these findings.

Today’s world has an increasing and faster trend towards the so-called “24-h societies”. To this we must add the need for continuous and necessary care that patients require, so it would be beneficial to apply preventive measures that minimize or avoid as much as possible these alterations in order to reduce the incidence of breast cancer among nurses.

## Figures and Tables

**Table 1:** Structure of the question using the PICO format (Patient/Problem/Population, Intervention/Indication, Comparison, Outcome).

P (Population)	Nurses
I (Intervention)	Work exposure: shift work hours
C (Comparison)	Breast cancer risk factors
O (Outcomes)	Level of association between risk factors (shift work schedule and breast cancer)

**Table 2:** Keywords. PICO format.

DeCS Term	MeSH Term
Horario de Trabajo por Turnos	Shift work schedule
Trastorno por trabajo a turnos	Shift work disorder
Neoplasia de mama	Breast neoplasms
Cáncer de mama	Breast cancer
Lactancia	Breast feeding
Enfermeras	Nurses
Enfermería	Nursing

DeCS: Descriptors in Health Sciences; MeSH: Medical Subject Headings.

**Table 3:** Items selected according to the established inclusion criteria.

Title	Date	Language	Study Type	Abstract	Meets Objective	Result
Does current scientific evidence support a link between light at night and breast cancer among female night-shift nurses? Review of evidence and implications for occupational and environmental health nurses [2].	YES	YES	YES	YES	YES	**☺**
Risk Factors for Breast Cancer, Including Occupational Exposures [3].	YES	YES	NO	YES	YES	☹
Case–control study of shift-work and breast cancer risk in Danish nurses, Impact of shift systems [4].	YES	YES	YES	YES	YES	**☺**
Circadian gene expression in peripheral blood leukocytes of rotating night shift nurses [5].	YES	YES	YES	YES	YES	**☺**
Night Work and Breast Cancer Risk Among Norwegian Nurses: Assessment by Different Exposure Metrics [6].	YES	YES	YES	YES	YES	**☺**
Mechanisms of breast cancer risk in shift workers, Association of telomere shortening with the duration and intensity of night work [7].	YES	YES	YES	YES	YES	**☺**
Breast Cancer Among Nurses Is the Intensity of Night Work Related to Hormone Receptor Status? [8]	YES	YES	YES	YES	YES	**☺**
Night shift work and other determinants of oestradiol, testosterone, and dehydroepiandrosterone sulphate among middle-aged nurses and midwives [9]	YES	YES	YES	YES	YES	**☺**
Night-shift work and breast and prostate cancer risk: updating the evidence from epidemiological studies [10]	YES	YES	YES	YES	NO	☹
Night-shift work and risk of breast cancer: a systematic review and meta-analysis [11]	YES	YES	YES	YES	NO	☹
Rotating Night Shift Work and Mammographic Density [12]	YES	YES	YES	YES	NO	☹
Rotating night shift work and polymorphism of genes important for the regulation of circadian rhythm [13]	YES	YES	YES	YES	YES	**☺**
Rotating night shift work and risk of breast cancer in the nurses’ health studies [14]	YES	YES	YES	YES	YES	**☺**
Systematic review of shift work and nurses’ health [15]	YES	YES	YES	YES	YES	**☺**
Association between light at night, melatonin secretion, sleep deprivation, and the internal clock: Health impacts and mechanisms of circadian disruption [16]	YES	YES	NO	YES	YES	☹
Does night work increase the risk of breast cancer? A systematic review and meta-analysis of epidemiological studies [20]	YES	YES	YES	YES	NO	☹
Rotating-shift nurses after a day off: peripheral clock genes’ expression, urinary melatonin, and serum 17-β-oestradiol levels [21].	YES	YES	YES	YES	YES	**☺**
Shift work and cancer risk: Potential mechanistic roles of circadian disruption, light at night, and sleep deprivation [22]	YES	YES	NO	YES	YES	☹
Shift work and cancer: State of Science and Practical Consequences [23]	YES	YES	YES	YES	NO	☹
Shift work, circadian gene variants and risk of breast cancer [24]	YES	YES	YES	YES	NO	☹
Sleep interruption associated with house staff work schedules alters circadian gene expression [25]	YES	YES	NO	YES	YES	☹
The Influence of Light at Night Exposure on Melatonin Levels among Canadian Rotating Shift Nurses [26]	YES	YES	YES	YES	NO	☹
Total and Cause-Specific Mortality of U.S. Nurses Working Rotating Night Shifts [27]	YES	YES	YES	YES	NO	☹
Sleep duration, nightshift work, and the timing of meals and urinary levels of 8-isoprostane and 6-sulfatoxymelatonin in Japanese women [28]	YES	YES	YES	YES	NO	☹
Night Shift Work, DNA Methylation and Telomere Length: An Investigation on Hospital Female Nurses [29]	YES	YES	YES	YES	YES	**☺**
TOTAL EXCLUDED RECORDS: 13						
TOTAL SELECTED ARTICLES: 12						

**☺** = Phase overcome/☹ = Phase not overcome.

**Table 4:** Methodological quality assessment outcomes through FLC 3.0. [19].

STUDY	Research Question: Is the Study Based on a Clearly Defined Research Question?	Method: Has the Study Method Allowed Minimizing Bias?	Results: Have the Outcomes Been Correctly Synthetized and Described?	Conclusions: Are the Conclusions Justified?	Conflict of Interests: Is the Existence or Absence of Conflict of Interests Well Described?	External Validity: Are the Study Outcomes Generalizable to the Population and Context of Interest?	Study Quality
Dickerman et al., 2012 [2]	Yes	Partially	Partially	Partially	Yes	Yes	INTERMEDIATE
Hansen et al., 2012 [4]	Yes	Partially	Yes	Partially	Yes	Partially	INTERMEDIATE
Reszka et al., 2013 [5]	Yes	Partially	Yes	Yes	Yes	Yes	INTERMEDIATE
Lie et al., 2011 [6]	Yes	Yes	Yes	Yes	Yes	Yes	HIGH
Erdem et al., 2017 [7]	Yes	Yes	Yes	Yes	Yes	Yes	HIGH
Lie et al., 2013 [8]	Yes	Partially	Yes	Yes	No information	Partially	INTERMEDIATE
Peplonska et al., 2016 [9]	Yes	Partially	Yes	Yes	Yes	Yes	INTERMEDIATE
Reszka et al., 2012 [13]	Yes	Partially	Yes	Yes	Yes	Yes	INTERMEDIATE
Wegrzyn et al., 2017 [14]	Yes	Partially	Yes	Yes	Yes	Yes	INTERMEDIATE
Rosa et al., 2019 [15]	Yes	Partially	Partially	Yes	Yes	Yes	INTERMEDIATE
Bracci et al., 2014 [21]	Yes	Partially	Yes	Yes	Yes	Yes	INTERMEDIATE
Carugno et al., 2019 [29]	Yes	Partially	Yes	Partially	Yes	Partially	INTERMEDIATE
**F.L.C 3.0 Platform Suggestions for Assessment.**							
		**“Method” Area: Yes**		**“Method” Area: Partially**		**“Method” Area: No**	
**Majority rest of areas: Yes**		High quality		Intermediate quality		Low quality	
**Majority rest of areas: Partially**		Intermediate quality		Intermediate quality		Low quality	
**Majority rest of areas: No**		Low quality		Low quality		Low quality	
*Not assessable*: Having answered ‘No information’ in the “Method” area or in “Majority of areas”, so assessing study quality is not possible							

**Table 5:** Characteristics of the studies included in the systematic review.

Author, Year, and Reference	Main Study Characteristics	Aim of the Study	Intervention and Instrument	Main Findings and Conclusions	Quality
Dickerman and Liu, 2012 [2]	Narrative review. Search terms: light at night, shift work, night shift, and breast cancer. Limits: English, human studies, publication after 2001. Critical appraisal tools are not specified.	To examine the impact of light at night exposure on breast cancer risk among female night-shift nurses, discuss possible mechanisms of action, and recommend future research and implications for practice.	Literature search in three databases. Reference lists at the end of found articles were also reviewed.	11 studies were found. Duration of Shift Work: studies reported a relationship between increased cancer risk and increased years and hours-per-week of night-shift work, compared with those who never worked at night. Rotating shifts: The risk of breast cancer may be proportional to the number of consecutive night shifts worked. Risk increases in permanent night shifts and long-term day-night rotating shifts. Light at night and melatonin levels have not been significantly associated in this study. Although the number of epidemiological studies is somewhat limited and further research is needed, evidence suggests that exposure to light during night shift work may increase the risk of breast cancer. Several potential mechanisms of action have been proposed: melatonin suppression, clock gene expression, sleep disruption, lifestyle factors, and lower vitamin D levels.	INTERMEDIATE
Hansen and Stevens, 2012 [4]	Case–control study: questionnaire. Sample: cohort of 58,091 female nurses (267 cases and 1035 controls)	To explore whether shiftwork causes breast cancer and which aspects of shiftwork are most problematic.	Telephone interview. Sociodemographic data were obtained. Questionnaire: years of schooling, occupational history and work schedule on cumulative years (day–evening, day–night, day–evening-night), tobacco, alcohol consumption, physical activity, reproductive history, use of hormone replacement therapy, occurrence of breast cancer in mother and/or sister.	Nurses who worked at night had a significantly longer working life, were younger at menarche and menopause, had fewer children, were older at the birth of their first child, spent fewer hours weekly on sport, and had slept fewer hours per night. Day–evening–night shiftwork is the most frequent rotating shift system. There is a tendency to increasing odds ratios for breast cancer by cumulative years of shiftwork and shift systems that disrupt circadian rhythms (i.e., night shifts and rotating day-night shifts).	INTERMEDIATE
Reszka et al., 2013 [5]	Cross-sectional study: questionnaire and gene expression analysis. Sample: 354 nurses and midwives currently working rotating night shifts and 370 nurses who work only during the day, all female.	To determine the effect of rotating night shift work on the expression of selected core circadian genes as indicators of peripheral clock.	Questionnaire: age, menopausal status, current job history (total years, years working at night and years without working at night [both from 0 to 15 years or more]), smoking, physical activity, quality of sleep, alcohol and antidepressants intake. Biological samples: Gene expression analysis was conducted among 92 pairs of nurses and midwives in the morning (6 a.m.–10 a.m.).	All the sample of this study had worked rotating night shifts in the past or during the study. An elevated circadian gene expression was observed among rotating night shift compared with day workers, influenced by the time of blood sampling. There was no association of the selected core genes of this study with the years working at night. The highest expression of a selected gene (Period1 - PER1) was found in nurses with longest lifetime duration of night shift.	INTERMEDIATE
Lie et al., 2011 [6]	Nested case and control study: telephonic structured questionnaire. Cohort: 49.402 Norwegian nurses. Cases: 699. Controls: 895.	To examine the relationship between shift work and breast cancer risk, including detailed evaluation of different exposure metrics of night-shift work.	Questionnaire: potential breast cancer risk factors (age, body mass index, menarche, menopause, hormonal therapy, alcohol and tobacco, breast cancer in mother/sister) and work-related factors (years of starting and ending employment, type of work site, radiographic procedures, type of work schedule [only days, only nights, both days and nights], years working at least 3 nights per month or rotating shifts, cumulative lifetime night shifts). Night shift: 12 p.m. until 6 a.m.	Previously identified risk factors for breast cancer are confirmed, for example, early menarche, lower number of childbirths, breast cancer in mother or sister, and hormonal treatment. Risk of breast cancer significantly increased among nurses who had worked for 5 years with ≥ 6 consecutive night shifts.	HIGH
Erdem et al., 2017 [7]	Nested case and control study: telephonic structured questionnaire and saliva samples for DNA extraction. Cohort: 49.402 Norwegian nurses. Cases: 699. Controls: 895.	To investigate telomere length variation as a potential mechanism of the association between long duration of night shift with consecutive nights and the increased risk of breast cancer.	Questionnaire: information on potential breast cancer risk factors and lifetime occupational history. Saliva samples were received from 563 cases and 619 controls. Telomere length was measured by polymerase chain reaction. Night shift: 12 p.m. until 6 a.m.	Telomere lengths were not significantly different in nurses that had worked night shifts compared with those that had worked only days. The shortening of telomeres is affected by intensive night work schedules and is associated with an increased risk of breast cancer among workers with long periods of consecutive night shifts., i.e., six consecutive nights over a period of more than 5 years.	HIGH
Lie et al., 2013 [8]	Nested case and control study: telephonic structured questionnaire. Cohort: 49.402 Norwegian nurses. Cases: 590. Controls: 757	To examine the relation between night work and hormone-receptor breast cancer subtypes (estrogen and progesterone).	Questionnaire: information on potential breast cancer risk factors and lifetime occupational history. Night shift: 12 p.m. until 6 a.m. Exposure measure: duration of work with a minimum of 6 consecutive night shifts. Information on the hormone receptor status of breast cancer cases was taken from the pathology reports submitted to the Cancer Registry for each cancer diagnosis.	A long duration (≥5 years) of night work with ≥6 consecutive night shifts was significantly associated with estrogen and progesterone positive tumors. The observed association between consecutive night shifts and positive progesterone receptor cancers suggests that progesterone could play an important role in the detrimental effects of night work.	INTERMEDIATE
Peplonska et al., 2016 [9]	Cross-sectional study: questionnaire and blood/urine collection. Sample: 594 female nurses and midwives; 345 premenopausal and 187 postmenopausal. Of them, 263 rotating night shifts and 269 day shifts.	To examine night shift work and body concentrations of sex hormones among pre- and postmenopausal women.	Questionnaire: information on potential breast cancer risk factors and lifetime occupational history (characteristics of night work: frequency of night shifts per month, duration of night shift work in years). Blood samples were collected in the morning (6 a.m.–10 a.m.). Night shift: 12 h of duration, from 7 p.m. to 7 a.m.	The most frequent working schedule was 6–7-night duties per month. There was no significant difference in the circulating sex hormone concentrations between current night shift workers and day workers. There was significant association between total duration of night work (>15 years) and higher estradiol levels among postmenopausal women. No significant associations were found with night work among premenopausal women, although the mean concentration of hormones is higher among women with longer night shift duration.	INTERMEDIATE
Reszka et al., 2012 [13]	Cross-sectional study: questionnaire and blood collection. Sample: 709 nurses and midwives; 348 in rotating shifts and 361 in non-rotating shifts.	To investigate the association between circadian genes polymorphisms and rotating night work adaptative mechanism.	Questionnaire: information on potential breast cancer risk factors and lifetime occupational history (characteristics of night work: frequency of night shifts per month, lifetime duration of night shift work in years). Blood samples (n = 709) were collected in the morning (6 a.m.–10 a.m.). Night shift: 12 h of duration, from 7 p.m. to 7 a.m.	There were no differences in clock genes (circadian) between nurses and midwives working on night and day rotating shifts. Differences were found in a specific genotype (cryptochrome 1) among nurses working long night shifts, as compared to those on the day shift, being more frequent in association with >8 night per month and >3 nights per week.	INTERMEDIATE
Wegrzyn et al., 2017 [14]	Case and control: questionnaires and medical records. Sample: 2 cohorts; NHS 78.516 women; NHS-2 114.559 women.	To examine the association between working on rotating night shifts and the risk of breast cancer on two prospective cohorts.	Questionnaire: lifestyle, occupational and environmental exposure, medication use, and medical condition. Rotating night shift work was defined as “3 or more night-shifts in one month”. Rotating shift work duration in prior years was assessed in a range from 0 years to >20 years. Medical records were consulted to confirm cancer diagnosis among nurses of the study.	Long-term rotating night work (>20 years) was associated with an increased risk of breast cancer among young women (ages 25–42) who accumulated night shifts in their early career. The median time to a first breast cancer event was of 13–14 years. Breast cancer risk by hormone receptor status was determined: associations with estrogen and progesterone positive tumors were significant for >20 years of cumulative shift work.	INTERMEDIATE
Rosa et al., 2019 [15]	Systematic review. Keywords: nurses, circadian rhythm, breast neoplasm, work schedule, among others. Dates: 2005–2016. Included: randomized control trials, observational studies and reviews. Limited to English language.	To describe the effects of shift work and desynchronization of circadian rhythms on nurse’s health.	Literature review in 5 databases. Quality assessment was performed.	24 articles were assessed. Shift work schedule causes physiological and psychological disturbances, also excessive fatigue, and interrupted sleep. Duration of shifts and number of consecutive nights are the main factors influencing sleep disorders. Rotating night shift work, stress and disruptions in circadian rhythms may lead to overweight and type 2 diabetes. A link between oestrogen, circulating melatonin, and breast cancer values is suggested. Risk of breast cancer was significantly higher in nursing staff working for >5 years with six consecutive night shifts.	INTERMEDIATE
Bracci et al., 2014 [21]	Cross-sectional study: questionnaire and blood/urine sampling. Sample: 60 female nurses with ≥ 2 years of rotating shifts and 56 female nurses with permanent day shifts	To compare levels of selected core clock genes expression, 6-sulfatoxymelatonine (aMT6s), and 17-β-oestradiol among workers in rotation shifts and day shifts after a day off.	Questionnaire: lifestyles, occupational and environmental exposures, medication use and chronotype (Morningness–Eveningness Questionnaire). Blood/urine samples: collected at 7 a.m. Gene expression, aMT6s, and estradiol levels were measured. Night shift: 10 p.m. to 7 a.m.	Significant expression of circadian genes was observed in shift workers. The influence of long-term shift work on circadian rhythm regulation is suggested, altering the expression of peripheral clock genes. Rotating shift participants did not show a significant difference in aMT6 levels but did show a significant difference in 17-β-oestradiol levels, as compared to day shift nurses.	INTERMEDIATE
Carugno et al., 2019 [29]	Cross-sectional study: questionnaire and blood sampling. Sample: 46 female nurses on night shift and 51 nurses working on morning shifts.	To analyze the association between night shift work (>2 years) and molecular alterations potentially related to increased carcinogenic risk.	Questionnaire: information on potential breast cancer risk factors and lifetime occupational history (focusing on shift work schedule and duration). Blood sample: extracted between 7:15 a.m. and 7:45 a.m. The analysis focused on DNA methylation of estrogen receptor genes, tumor suppressor genes, and telomere length.	DNA methylation of oestrogen receptor genes (ESR1, ESR2) play a significant role in the proliferation of breast tissue stimulated by estrogens, which is a known as breast cancer risk factor. Reduced telomere length is found in nurses with at least 12 years of night shifts.	INTERMEDIATE

## Supplementary Materials

The following are available online at https://www.mdpi.com/1010-660X/56/12/680/s1, Table S1. Search strategy in databases.
