# Supplementary material for: Relationship between Night Shifts and Risk of Breast Cancer among Nurses: A Systematic Review
Source: Medicina (Kaunas). 2020 Dec 10;56(12):680. doi: 10.3390/medicina56120680 (PMC7764664; doi:10.3390/medicina56120680)
Supplement: Supplementary file 1 [file medicina-56-00680-s001.pdf]

Table S1. Search strategy in databases.

Database	Date	Search strategy	Total articles	Pre-selected articles
Cochrane	07/07/2020	#1 shift work disorder<br>#2 breast neoplasms<br>#3 breast cancer<br>#4 nurse<br>#5 nursing<br>#6 (#1) AND (#2 OR 3#) AND (4# OR 5#) with Cochrane Library publication date Between Jul 2010 and Jul 2020, in Cochrane Reviews.	196	0
Pubmed	07/07/2020	Search: shift work schedule AND (breast neoplasms OR breast cancer) AND (nurse OR nursing) Filters: Clinical Trial, Meta-Analysis, Randomized Controlled Trial, Review, Systematic Review, in the last 10 years Sort by: Most Recent<br>((("shift work schedule"[MeSH Terms] OR (("shift"[All Fields] AND "work"[All Fields]) AND "schedule"[All Fields])) OR "shift work schedule"[All Fields]) AND ((("breast neoplasms"[MeSH Terms] OR ("breast"[All Fields] AND "neoplasms"[All Fields])) OR "breast neoplasms"[All Fields]) OR (((("breast neoplasms"[MeSH Terms] OR ("breast"[All Fields] AND "neoplasms"[All Fields])) OR "breast neoplasms"[All Fields]) OR ("breast"[All Fields] AND "cancer"[All Fields])) OR "breast cancer"[All Fields]))) AND ((((((((((((("nurse s"[All Fields] OR "nurses"[MeSH Terms]) OR "nurses"[All Fields]) OR "nurse"[All Fields]) OR "nurses"[All Fields]) OR "nursing"[MeSH Terms]) OR "nursing"[All Fields]) OR "nursings"[All Fields]) OR "nursing"[MeSH Subheading]) OR "breast feeding"[MeSH Terms]) OR ("breast"[All Fields] AND "feeding"[All Fields])) OR "breast feeding"[All Fields]) OR "nursing s"[All Fields]) OR ((((((((((("nurse"[All Fields] OR "nurses"[MeSH Terms]) OR "nurses"[All Fields]) OR "nurse"[All Fields]) OR "nurses"[All Fields]) OR "nursing"[MeSH Terms]) OR "nursing"[All Fields]) OR "nursings"[All Fields]) OR "nursing"[MeSH Subheading]) OR "breast feeding"[MeSH Terms]) OR ("breast"[All Fields] AND "feeding"[All Fields])) OR "breast feeding"[All Fields]) OR "nursing"[All Fields]))	29	7

CINAHL	08/07/2020	Shift work schedule AND ((breast neoplasms OR breast cancer)) AND ((nurse OR nursing)). Limits – Available abstract; Publication date: 20100701-20200731. Expanders - Apply equivalent subjects. Search modes - Booleano/Frase.	1	1
Web of Science	08/07/2020	SUBJECT: ((shift work schedule) AND (breast neoplasms OR breast cancer) AND (nurse OR nursing)). Refined by: Open access: (OPEN ACCESS) Time period: 2010-2020. Indexes: SCI-EXPANDED, SSCI, A&HCI, CPCI-S, CPCI-SSH, BKCI-S, BKCI-SSH, ESCI, CCR-EXPANDED, IC.	22	9
Science Direct	08/07/2020	ALL FIELDS Shift work schedule AND (breast neoplasms OR breast cancer) AND (nurse OR nursing) AND occupational risk factor AND PUBYEAR > 2010.<br>Type of document: Review articles (62) and Research articles (44)	106	7
Scopus	08/07/2020	TITLE-ABS-KEY (((shift AND work AND schedule) Y (mama y neoplasias o mama y cáncer) Y (enfermera o enfermería)) Y (LIMIT-TO (ACCESSTYPE(OA))) Y (LIMIT-TO (PUBYEAR, 2019) O LIMIT-TO (PUBYEAR, 2018) O LIMIT-A (PUBYEAR, 2017) O LIMIT-TO (PUBYEAR, 2016) O LIMIT-TO (PUBYEAR, 2015) O LIMIT-TO (PUBYEAR , 2014) O LIMIT-TO (PUBYEAR , 2013) O LIMIT-TO (PUBYEAR , 2012) O LIMIT-TO (PUBYEAR , 2011) O LIMIT-TO (PUBYEAR , 2010)) Y (LIMIT-TO (DOCTYPE, "ar") O LIMIT-TO (DOCTYPE, "re re")) Y (LIMIT-TO (EXACTKEYWORD, "Breast Cancer") O LIMIT-TO (EXACTKEYWORD , "Nurse") O LIMIT-TO (EXACTKEYWORD , "Shift Worker") O LIMIT-TO (EXACTKEYWORD , "Circadian Rhythm"))	16	12
Dialnet	27/07/2020	Trabajo por turnos AND cáncer de mama. Type: journal article. Years range 2010 – 2019. Descriptors: breast cancer, circadian disruption, shift work; cáncer de mama, disrupción circadiana, trabajo por turnos.	1	1
TOTAL PRE-SELECTED ARTICLES				37
TOTAL ARTICLES AFTER DUPLICATES REMOVAL				25
